# The European Bioinformatics Institute (EMBL-EBI) in 2021

**DOI:** 10.1093/nar/gkab1127

**Published:** 2021-11-25

**Authors:** Gaia Cantelli, Alex Bateman, Cath Brooksbank, Anton I Petrov, Rahuman S Malik-Sheriff, Michele Ide-Smith, Henning Hermjakob, Paul Flicek, Rolf Apweiler, Ewan Birney, Johanna McEntyre

**Affiliations:** European Molecular Biology Laboratory, European Bioinformatics Institute (EMBL-EBI), Wellcome Genome Campus, Hinxton, Cambridge CB10 1SD, UK; European Molecular Biology Laboratory, European Bioinformatics Institute (EMBL-EBI), Wellcome Genome Campus, Hinxton, Cambridge CB10 1SD, UK; European Molecular Biology Laboratory, European Bioinformatics Institute (EMBL-EBI), Wellcome Genome Campus, Hinxton, Cambridge CB10 1SD, UK; European Molecular Biology Laboratory, European Bioinformatics Institute (EMBL-EBI), Wellcome Genome Campus, Hinxton, Cambridge CB10 1SD, UK; European Molecular Biology Laboratory, European Bioinformatics Institute (EMBL-EBI), Wellcome Genome Campus, Hinxton, Cambridge CB10 1SD, UK; European Molecular Biology Laboratory, European Bioinformatics Institute (EMBL-EBI), Wellcome Genome Campus, Hinxton, Cambridge CB10 1SD, UK; European Molecular Biology Laboratory, European Bioinformatics Institute (EMBL-EBI), Wellcome Genome Campus, Hinxton, Cambridge CB10 1SD, UK; European Molecular Biology Laboratory, European Bioinformatics Institute (EMBL-EBI), Wellcome Genome Campus, Hinxton, Cambridge CB10 1SD, UK; European Molecular Biology Laboratory, European Bioinformatics Institute (EMBL-EBI), Wellcome Genome Campus, Hinxton, Cambridge CB10 1SD, UK; European Molecular Biology Laboratory, European Bioinformatics Institute (EMBL-EBI), Wellcome Genome Campus, Hinxton, Cambridge CB10 1SD, UK; European Molecular Biology Laboratory, European Bioinformatics Institute (EMBL-EBI), Wellcome Genome Campus, Hinxton, Cambridge CB10 1SD, UK

## Abstract

The European Bioinformatics Institute (EMBL-EBI) maintains a comprehensive range of freely available and up-to-date molecular data resources, which includes over 40 resources covering every major data type in the life sciences. This year's service update for EMBL-EBI includes new resources, PGS Catalog and AlphaFold DB, and updates on existing resources, including the COVID-19 Data Platform, trRosetta and RoseTTAfold models introduced in Pfam and InterPro, and the launch of Genome Integrations with Function and Sequence by UniProt and Ensembl. Furthermore, we highlight projects through which EMBL-EBI has contributed to the development of community-driven data standards and guidelines, including the Recommended Metadata for Biological Images (REMBI), and the BioModels Reproducibility Scorecard. Training is one of EMBL-EBI’s core missions and a key component of the provision of bioinformatics services to users: this year's update includes many of the improvements that have been developed to EMBL-EBI’s online training offering.

## INTRODUCTION

Based in Hinxton (Cambridge, UK), EMBL-EBI maintains the world's most comprehensive range of freely available and up-to-date molecular data resources. EMBL-EBI’s activities take place within the broader context of service provision at EMBL, which enables scientists from around the world to take advantage of a broad portfolio of world-class infrastructures and resources through a single Europe-wide partner.

EMBL-EBI has a 5-fold mission, including providing scientific services and training, carrying out bioinformatics research, disseminating cutting-edge technologies to industry, and supporting the coordination of biomolecular data provision in Europe. The synergy between these areas is one of the Institute's unique strengths. EMBL-EBI hosts a suite of over 40 open data resources, which encompass every data type in molecular biology. Data resources include deposition databases (which archive experimental data) and added-value databases (which add value to archived data by providing annotation, curation, reanalysis and integration), as well as open-source software tools. EMBL-EBI services are provided collaboratively, and many of its data resources are run in partnership with other organisations all over the world.

EMBL-EBI services are crucial to the life science community. On an average day in 2020, EMBL-EBI resources received over 81 million web and API requests. Throughout 2020, EMBL-EBI services were visited via almost 41 million IP addresses (the number of IP addresses is an indication of the number of users, but not an exact count), and over 545,000 IP addresses visited its Train online platform. Moreover, by the end of 2020 EMBL-EBI reached over 390 petabytes of raw data storage. An economic Value and Impact study commissioned by EMBL-EBI in 2020 on its open data resources estimated a use value of £5.5 billion per annum for EMBL-EBI resources (calculated by measuring the value is the time users spend using EMBL-EBI managed data resources) (www.embl.org/documents/document/embl-ebi-impact-report-2021). The same study highlighted research impacts worth £1.3 billion annually that could not have been realised in the absence of EMBL-EBI managed resources. These figures show the importance of EMBL-EBI managed resources for the global life sciences community, and more generally they demonstrate how open data resources are central to scientific progress.

**Figure 1.**
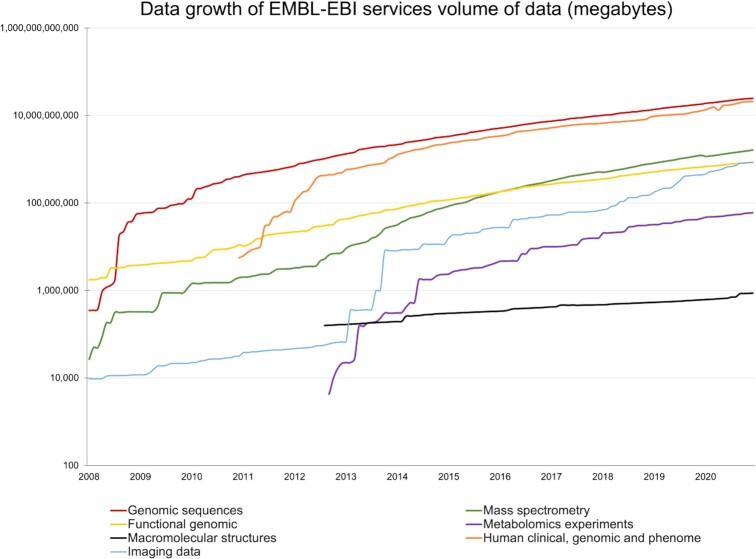
Data growth of EMBL-EBI services by data type. Y axis in logarithmic scale.

In 2020, data submissions to EMBL-EBI from researchers around the world have continued to grow (Figure 1). EMBL-EBI hosts 9 data deposition resources for: genomic sequence data (European Nucleotide Archive), controlled access human genomic and phenomic data consented for research use (European Genome-phenome Archive), mass spectrometry data (PRoteomics Identification DatabasE), functional genomic data (ArrayExpress), metabolomic data (MetaboLights), data on macromolecular structures (PDBe), electron cryo-microscopy and biological imaging data (EMDB, BioImage Archive and EMPIAR).

In recent years, we have seen a large increase in the deposition of biological imaging data into the BioImage Archive (1), the Electron Microscopy Data Bank (EMDB; (2)), and the Electron Microscopy Public Image Archive (EMPIAR;(3)). The deposition volume of imaging data for 2020 alone surpassed all previous annual depositions by almost 20%. The EMBL-EBI data resource portfolio has been hosting imaging data since the early 2000s and has been evolving ever since to keep up with the development of the field. EMDB was established in 2002 and hosts electron cryo-microscopy maps and tomograms of macromolecular complexes and subcellular structures. In 2016, EMPIAR was launched in response to the electron cryo-microscopy (cryo-EM) community's need for public archiving of raw 2D image data. Since then, EMPIAR has expanded its remit to also archive data from various volume-EM and X-ray tomography methods: in June 2021, EMPIAR celebrated its first petabyte of image data. With the launch of the BioImage Archive in 2019, EMBL-EBI’s remit was expanded to include all reference bioimaging data. EMBL-EBI is committed to ensuring growing research data are open and available through continuous transformation and maintenance of its data resource portfolio.

Over the past year, EMBL-EBI has undergone a number of important changes. This paper will outline some of the most significant updates in EMBL-EBI services and training in 2021.

## SERVICES UPDATES

### New resources and collaborations

EMBL-EBI’s mission to ‘provide freely available data and bioinformatics services to all facets of the scientific community in ways that promote scientific progress’ is closely tied to its commitment to maintain a data resource portfolio that is responsive to the needs of the research communities it serves. In 2021, EMBL-EBI launched two new collaboratively built resources.

EMBL-EBI launched the AlphaFold Protein Structure Database, AlphaFold DB (https://www.alphafold.ebi.ac.uk/), in collaboration with the artificial intelligence company DeepMind. AlphaFold DB provides open access to the predicted protein structures for the entire human proteome, alongside that of 20 other organisms important to biomedical research, including mice, rats, *Caenorhabditis elegans* and *Drosophila melanogaster*. The initial release of AlphaFold DB contained over 350,000 structures. The predictions are generated using AlphaFold 2, an artificial intelligence (AI) system developed by DeepMind (4) and applied to the whole human proteome (5). The protein-structure predictions in AlphaFold DB will enable new opportunities for structural biology research, structural prediction research, and structural bioinformatics research, as well as for other life-sciences disciplines, such as drug discovery (https://www.ebi.ac.uk/about/news/opinion/alphafold-potential-impacts). An extensive description of AlphaFold DB and its features is available in this NAR Database Issue at (Varadi *et al.*, 2022).

PGS Catalog (https://www.pgscatalog.org/) is an open resource for polygenic scores. A polygenic score (PGS) is a measurement for genetic predisposition for a trait or phenotype that aggregates the effects of many genetic variants into a single number.

A single PGS may encompass hundreds-to-millions of trait-associated genetic variants, owing to increasingly powerful genome-wide association studies (GWAS). PGS are increasingly used in the study of various diseases, including heart disease, cancer and psychiatric illnesses. The PGS Catalog was instantiated to respond to a community need to systematically evaluate PGS and provide access to the information necessary to calculate the scores themselves (6,7). The Catalog provides an open community platform for PGS research, and enables deposition of data from users, expert curation of uploaded data and for programmatic access. As of August 2021, the Catalog included 826 polygenic scores for 214 different traits linked to 207 publications. While the Catalog includes information for both general traits and diseases, most of the data deposited so far is in relation to pathologies. Importantly, the establishment of PGS Catalog was matched with new efforts in setting specific data standards for PGS data, resulting in the establishment of the Polygenic Risk Score Reporting Standards (PRS-RS) by a group of researchers from institutions around the world, including EMBL-EBI (6).

### Updates and new features to existing resources

All EMBL-EBI data resources are continuously updated to ensure that they are relevant for the research community. EMBL-EBI databases that have had major updates are detailed in articles elsewhere in this issue of Nucleic Acids Research and include Ensembl (8), Ensembl Genomes (9), the Ensembl COVID-19 Browser (10), the European Variation Archive (EVA) (11), the European Genome-phenome Archive (EGA) (12), Expression Atlas (13), Complex Portal (14), IntAct (15), Reactome (16), and PRIDE (17). Here, we highlight some of the major developments that have been carried out over the last year in a number of other resources.

- **The European COVID-19 Data Platform**

In March 2020, EMBL-EBI, supported by the European Commission and other funders, set to work on building the European COVID-19 Data Platform (https://www.covid19dataportal.org/), which was launched in April 2020 (18). The COVID-19 Data Platform is the result of collaboration between many partners. It was intended to be a resource to link the clinical, epidemiological, and public health worlds in response to the SARS-CoV-2 pandemic. Since its launch, the European COVID-19 Data Platform has greatly evolved, to keep up with the work of the European scientific community and to better support data provision around the pandemic. 

**Figure 2.**
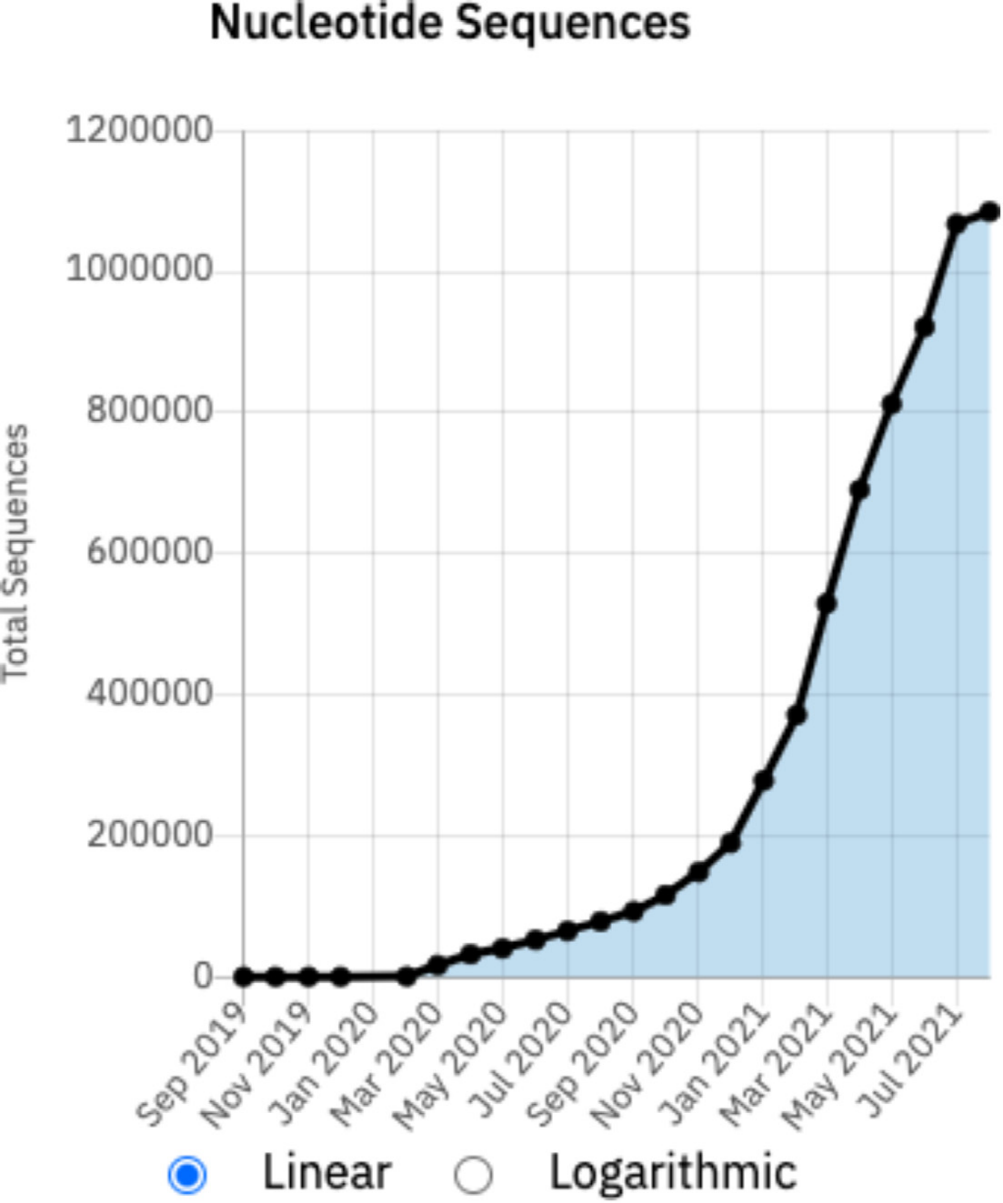
Growth of SARS-CoV-2 nucleotide sequence submissions to the COVID-19 Data Platform.

During its first year, the European COVID-19 Data Portal received over 4.5 million requests by over 180,000 users. During the same period of time, users accessed over 1.5 million records, including viral genomic sequences, SARS-CoV-2 protein entries, and gene and protein expression data of human genes implicated in the virus infection of the host cells. Moreover, since March 2020 users of the Portal have submitted over 2.2 million datasets, spanning across the various data types present in the COVID-19 Data Portal, in addition to > 450,000 linked publications. This includes >1.1 million assembled SARS-CoV-2 nucleotide sequences (Figure 2) and ∼1.1 million raw SARS-CoV-2 reads, the growth of which can be tracked via the Statistics page (https://www.covid19dataportal.org/statistics).

At the time of writing, the COVID-19 Data Portal amalgamated data on SARS-CoV-2 and COVID-19 from 20 data resources at EMBL-EBI. The COVID-19 Data Portal itself has been improved over the past year, with the addition of a number of new features, including an FAQ section, phylogenetic tree representation of variants, and highlighting of the two main reference sequences for SARS-CoV-2. Moreover, the Portal now includes a bulk download tool, through which users can download, and synchronise with, large quantities of data.

The European COVID-19 Data Platform has promoted the creation of national data portals, which provide information, guidelines, tools and services to support researchers in creating and sharing research data on COVID-19. The purpose of the national data portals is to showcase and highlight COVID-19 research data from each of the participating countries, to provide scientists working within these countries access to initiatives and resources organised at the national level and to allow connectivity with the central COVID-19 Data Portal for the broader data and services that it provides. Similarly to the broader European COVID-19 Data Portal, the national data portals rely on open and FAIR access of sequence data, and are regularly updated with new tools, services and data. At the time of writing, there were 10 national data portals, including portals for Italy, Poland, Sweden, Slovenia, Turkey, Norway, Spain, Greece, Estonia and Japan. National data portals were set up using a set of tools provided by EMBL-EBI and are connected by the COVID-19 National Data Portal network. A number of SARS-CoV-2 Data Hubs have been assigned corresponding to national SARS-CoV-2 surveillance efforts and a number are particularly active across the breadth of data management and analysis services on offer. Greece, for example, utilises integrated workflows to generate consensus sequence and variant calling data from analysis of submitted raw reads.

The SARS-CoV-2 sequence data that were submitted to the European COVID-19 Data Platform was subsequently used by the Versatile Emerging infectious disease Observatory (VEO) to produce its reports on mutations and variation in SARS-CoV-2 to a large number of public health and other stakeholders. The most recent VEO report at the time of writing, its seventh, is available at https://www.covid19dataportal.org/news/latest-veo-report-published?nid=1888.

We are keen to acknowledge the strong partnerships that have enabled EMBL-EBI to respond rapidly and at scale with the European COVID-19 Data Platform, in particular EMBL-EBI’s partnership with ELIXIR. Through its network of bioinformatics experts and capacity around Europe, ELIXIR has been particularly important in the mobilisation of European data activities and National Portals.

- **Europe PMC is now offering access to preprints**

Europe PMC provides access to a worldwide collection of life science literature (https://europepmc.org/). Since 2018 Europe PMC has been aggregating and indexing preprint abstracts alongside peer-reviewed full text articles (19). Preprints in Europe PMC are enriched with links to journal published versions, open peer review materials, related data and other useful resources. There are now over 336,000 preprints indexed in Europe PMC. Since July 2020 Europe PMC has been making the full text of COVID-19 preprints available for search, reading and reuse in standard JATS XML format.

At the time of writing, over 34,000 full text COVID-19 preprints were available in addition to open COVID-19 peer-reviewed articles. Preprints in EuropePMC are indexed from many servers, including medRxiv, bioRxiv, arXiv, ChemRxiv, SSRN and Research Square. Indexing the full text of preprints (rather than just abstracts) makes them significantly more discoverable. The COVID-19 full text preprint collection can also be accessed programmatically, with over 20,000 open access preprints available via bulk download as of September 2021. This enables large-scale analyses of the COVID-19 preprint corpus and provides a basis to explore new approaches to open and rapid publication systems.

- **EBI Search has grown and further developed its Search-as-a-Service features**

EBI Search (20) is a search engine that provides easy and uniform access to EMBL-EBI data resources (https://www.ebi.ac.uk/ebisearch/s4). Through EBI Search, users can navigate across different data resources via a network of cross-references. EBI Search is maintained in sync with all established EMBL-EBI Data Resources and is currently indexing ∼5 billion entries.

EBI Search can be accessed in a web browser or programmatically using the RESTful Web Services interface. The EBI Search API (also known as ‘EBI Search as a Service’) is being used behind the scenes by a growing number of EMBL-EBI data resources and tools, including the COVID-19 Data Portal, Ensembl, RNAcentral, OmicsDI, ENA, MGnify, InterPro, and the HMMER web server. Programmatic use of EBI Search allows it to be used in analytical pipelines and workflows, enriching the results from analysis tools (20), such as BLAST + and InterProScan. These have been heavily used during the SARS-CoV-2 pandemic, averaging 1.4 million jobs per day.

- **trRosetta and RoseTTAfold models were introduced in Pfam and InterPro**

In March 2021 the Pfam and InterPro (http://pfam.xfam.org/; https://www.ebi.ac.uk/interpro/) databases made available a set of 6370 structural models created by researchers at the University of Washington using the trRosetta software (21). These models were added prior to the release of the 365,000 AlphaFold models that have been released in AlphaFold DB. Although the trRosetta models are not quite as accurate as DeepMind's AlphaFold 2 predictions, they are certainly of a high-enough quality for many applications.

**Figure 3.**
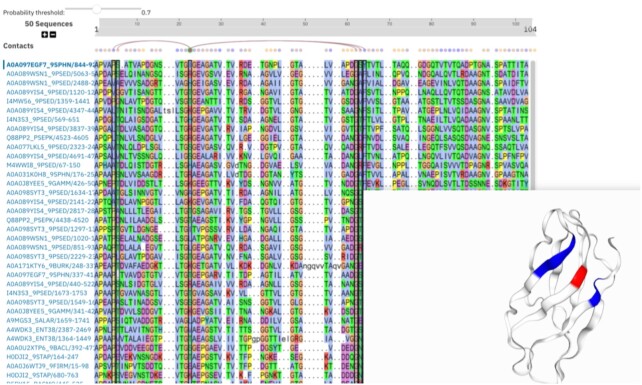
Contact map and structural model for PF17936 as seen through the InterPro website.

The trRosetta models in Pfam and InterPro were created using large sequence alignments of UniProtKB sequences from the Pfam resource. The addition of these trRosetta structural models substantially increased the proportion of Pfam families with structural information from 53% to 88% (data from Pfam 33.1). To enable users to view and interpret the structural models, the team developed a new interface within the InterPro website, under the trRosetta tab of Pfam families (see Figure 3). This interface allows users to see which residues in the Pfam seed alignment are predicted by trRosetta to be close in space. An archive containing all of the trRosetta models and contact maps can be downloaded from the Pfam and InterPro FTP sites. The structural models are particularly useful within Pfam, for example to understand when a Pfam family is part of a larger superfamily, or clan as they are called in Pfam. They also improve annotations, as researchers can determine domain boundaries more precisely. Through the identification of distant homologues, relationships and functionalities can be predicted for proteins for which there is scarce information. These new models, visualisations and improvements of Pfam families are beneficial for the community and help Pfam to expand its coverage.

Since the trRosetta structural models were incorporated into Pfam and InterPro, a new prediction method called RoseTTAfold has been released (22), which is more accurate than trRosetta. RoseTTAfold was inspired by the AlphaFold 2 system, and it can generate accurate structural predictions based on alignments with few sequences, which was not the case for trRosetta. RoseTTAfold models are currently available through the Pfam FTP site and will replace the trRosetta models in the Pfam and InterPro web interfaces. The addition of AlphaFold models to the Pfam and InterPro interfaces further improves structural coverage of these resources. In particular, because the AlphaFold models are for full length proteins they can add crucial structural information for the inter domain regions and their relative orientations.

- **RNAcentral developed R2DT**

**Figure 4.**
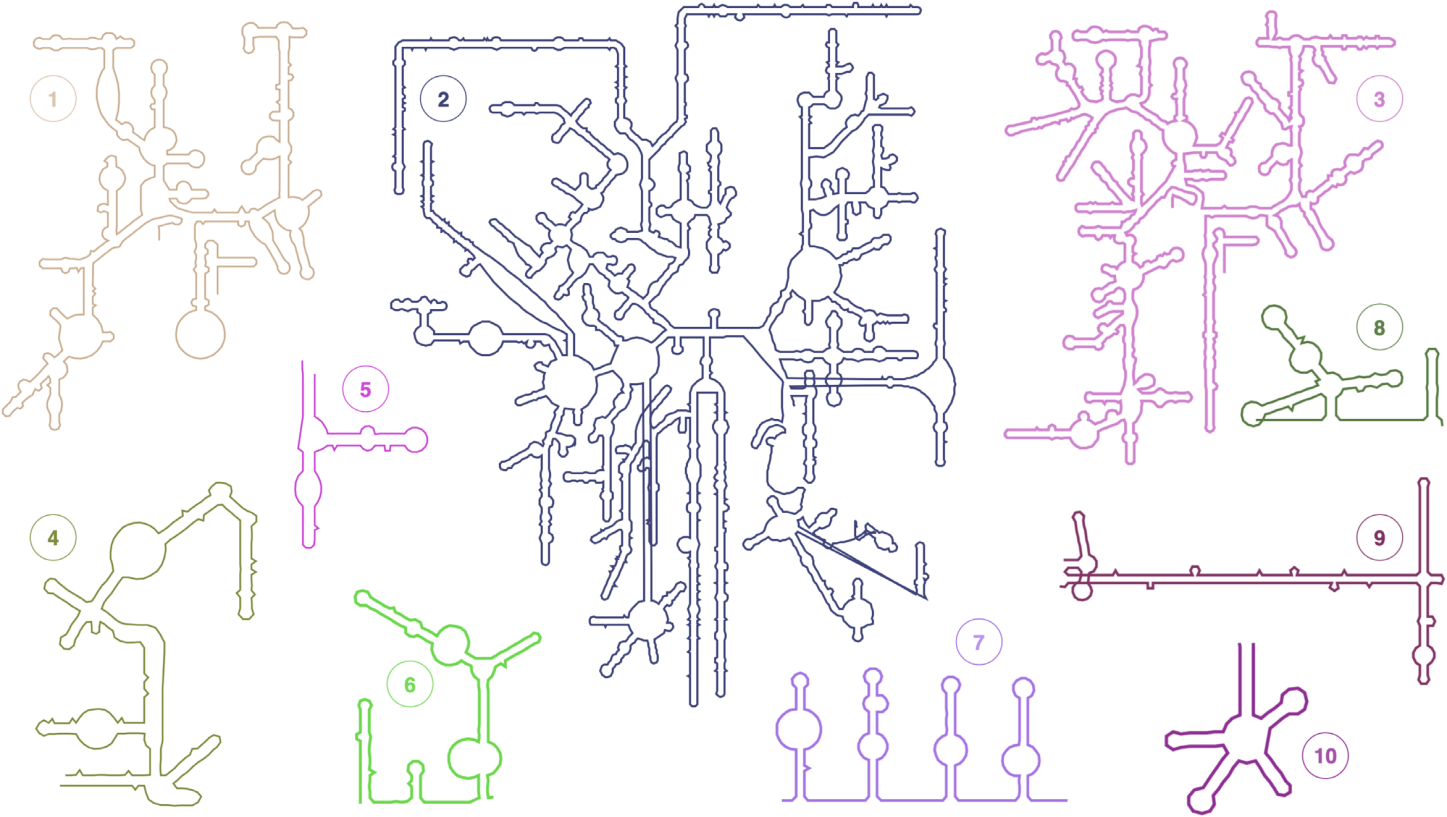
Example secondary structure diagrams for human RNAs produced by R2DT (shown in simplified layouts). 1) mitochondrial small ribosomal subunit RNA; 2) large ribosomal subunit RNA; 3) small ribosomal subunit RNA; 4) RNAse P; 5) 5S ribosomal RNA; 6) SNORD3A RNA; 7) SCARNA13 RNA; 8) U1 snRNA; 9) 7SL RNA; 10) selenocysteine tRNA.

RNAcentral (https://rnacentral.org) is the world's largest database of non-coding RNA sequences (23). RNAcentral has recently expanded to incorporate the visualisation of the predicted secondary structure for its RNA sequences by using a new method called R2DT (24). While numerous approaches exist for visualising RNA secondary structure, R2DT is the only tool capable of automatically displaying RNAs using the standard orientations that are commonly used by the RNA community. This facilitates comparative structural analysis between R2DT predictions and other RNA structure data. To generate its visualisations, R2DT uses a library of over 4000 templates, including a wide range of structured RNAs, from tRNAs to the large ribosomal RNAs (Figure 4).

Given an input sequence, R2DT automatically selects the best matching template and uses it for generating a secondary structure diagram, while accommodating any sequence-specific features, such as insertions or deletions. At the time of writing, R2DT had precomputed the secondary structures for >20 million RNAcentral sequences. Users can also analyse their own sequences using an R2DT webserver (https://rnacentral.org/r2dt) or run R2DT locally. In addition, R2DT is integrated into the sequence similarity search deployed on the RNAcentral and Rfam websites (25).

- **UniProt and Ensembl launched Genome Integrations with Function and Sequence (GIFTS)**

In 2021, the Uniprot and Ensembl teams launched the Genome Integrations with Function and Sequence (GIFTS) service, which aims to provide a common framework for Ensembl and UniProt entity mappings. GIFTS (https://www.ebi.ac.uk/gifts/) connects Ensembl genes and transcripts to UniProt protein sequences: through its user-friendly platform, researchers can access human and mouse data and connect genomic and protein information. At the time of writing, GIFTS mapped 99% of UniProtKB human canonical sequences. Through GIFTS, biocurators at EMBL-EBI have the tools to annotate and exchange transcript and protein sequence information to map content between Ensembl and UniProt, improving the consistency of annotation available to users of these services.

### Setting and implementing standards to drive FAIR data

EMBL-EBI is committed to ensuring the data it hosts are as Findable, Accessible, Interoperable and Reusable (FAIR) as possible (26). The process of making data FAIR can be enabled by the development of community-driven data standards and guidelines, and by their integration with open data resources. EMBL-EBI service teams engage directly with the research communities they serve in order to develop new guidelines and standards as required. In 2021, EMBL-EBI has contributed to the development of three new sets of data guidelines: the Polygenic Risk Score Reporting Standards (PRS-RS) as described above, the Recommended Metadata for Biological Images (REMBI), and the BioModels Reproducibility Scorecard.

REMBI, Recommended Metadata for Biological Images (27), was developed together with collaborators from over 30 institutions worldwide with the aim to maximise the potential for reuse of bioimaging data. In order for bioimaging data to fulfil their potential, it is essential for them to be systematically archived in public databases and to be accompanied by standardised metadata. REMBI proposes guidelines for such metadata, aiming to address the needs of all biological imaging communities that use light and electron microscopy. Metadata covered by REMBI (https://bit.ly/rembi_v1) have been divided into eight categories, including study information, study components (e.g. imaging method), biosample information (e.g. species being imaged and experimental variables), specimen data (e.g. preparation method), image acquisition (e.g. acquisition parameters), image data (e.g. format and compression), image correlation (e.g. related images and their relationship), and analysed data (e.g. analysis method and details). The BioImage Archive is adopting REMBI for its submission pipeline: submitters will be guided through the process of supplying metadata for their imaging datasets, supported by clear examples and explanations at each stage. This process will provide integration with other existing data resources, for example by sharing sample metadata with BioSamples (28). REMBI will incorporate/support detailed community specifications where they exist, such as the 4DN-BINA-OME standard for light microscopy (29). The implementation of REMBI into BioImage Archive will improve findability and reuse of image data, to better unlock the potential of the BioImage Archive's collections.

The BioModels reproducibility Scorecard was developed to address reproducibility issues in systems biology. Reproducibility of scientific results is an important concern in various fields of research, including systems biology modelling. In systems biology, models are a set of mathematical equations representing biological systems that are used to study emergent systems behaviour. BioModels (https://www.ebi.ac.uk/biomodels/) is the repository of systems biology models managed by EMBL-EBI (30).

Mathematical equations are generally expected to consistently reproduce the results they have generated in the past, unlike experiments that can be affected by several confounding factors. However, increasing concern in the modelling community indicated that reproducibility may be an issue for mathematical modelling as well as for experimental work. The team at BioModels therefore performed one of the largest reproducibility studies (31) to date to assess the reproducibility of system biology modeling. The study included 455 mathematical models from peer-reviewed research articles published in 152 different life science journals. This analysis revealed that about half (49%) of the models could not be reproduced using the information provided in the manuscript. 12% of the models could be reproduced with further efforts (e.g. empirical correction or contacting corresponding authors). However, the remaining 37% of the models remained non-reproducible.

To address this crisis, the BioModels team has proposed a reproducibility scorecard (https://www.ebi.ac.uk/biomodels/reproducibility (31)) with eight simple ‘yes’ or ‘no’ questions related to the model sharing. The scorecard can be used by journal reviewers and editors during the peer-review process. A ‘yes’ answer to each question gains a score of 1: therefore, a model could get up to a score of 8. In addition to recommending a wider community adaptation, the team plans to implement the scorecard in BioModels in the model submission pipeline.

## UPDATES TO EMBL-EBI TRAINING

**Figure 5.**
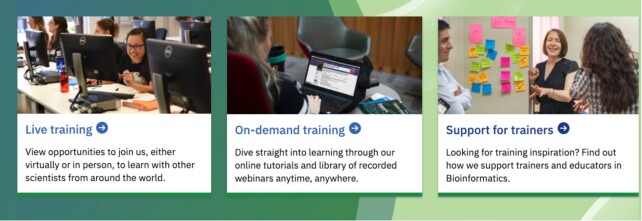
EMBL-EBI’s three training channels.

Training is one of EMBL-EBI’s core missions and a key component of the provision of bioinformatics services to users (www.ebi.ac.uk/training). External training activities across all parts of EMBL-EBI are coordinated with the goal of creating a coherent, high-quality programme with global reach. An ongoing project involving the EMBL-EBI web development team to make training content FAIR (32), is making rapid progress and the new training website was launched in February 2021. Major improvements include the following:

- EMBL-EBI’s entire collection of courses, webinars and online tutorials is fully searchable using a free-text search based on EMBL-EBI’s Search (20). Content can also be accessed through three channels - live, on-demand and trainer support (Figure 5). This reflects how the programme has been developed to make it resilient to the pandemic.
- The ‘live’ channel provides access to webinars, full-length virtual courses, and in-person courses, which will be reintroduced in 2022 whilst retaining a lively virtual programme. Events in the live channel are all time-bound and require prior application or registration. Course participants are given exclusive access to an online handbook before the start of the course and for six months afterwards; this includes information private to the cohort of participants on that specific instance of the course. Once the course has finished, the Training team creates an openly accessible and searchable set of course materials.
- The ‘on-demand’ channel provides free and open access to pre-recorded webinars and online tutorials. This collection is extensive: as of August 2021 it included 97 webinars and 80 tutorials. Here, users can learn about the methods used to generate, process and analyse biological data, and learn how to make full use of EMBL-EBI’s rich and varied data resources. Tutorials are included on topics of broad interest to data-driven bioscientists, including data management and ELSA topics.
- The ‘Support for Trainers’ channel summarises how bioinformatics educators and trainers can make full use of EMBL-EBI training content, gives guidance on how to work with us to create tailored courses for a specific audience, provides information on ‘train the trainer’ activities with EMBL-EBI (33), and provides a gateway to the Competency Hub (https://competency.ebi.ac.uk/). This provides open access to competency frameworks of relevance to the molecular life sciences, enabling learning professionals to determine the needs of their audiences and analyse training gaps. The Competency Hub provides a stable, version-controlled home for the ISCB’s Competency Framework (34) and for several other project-based frameworks.
- Several ‘behind the scenes’ improvements contribute to the new site's adherence to FAIR principles. Bioschemas tags for live courses and webinars enable EMBL-EBI Training content to be pulled by other aggregators, such as ELIXIR’s Training e-Support System. Course information can also seamlessly be transferred from the training content database to the new embl.org events page (www.embl.org/events).
- The authoring tool for self-paced tutorials has been re-developed. The new WordPress-based authoring tool streamlines the writing, publishing and updating process and brings it in line with the content authoring mechanism for EMBL team websites. Finally, the EMBL-EBI training website was an early adopter of EMBL’s open Visual Framework (https://stable.visual-framework.dev/); this has enabled the Training team to achieve a clear visual identity for all of EMBL’s external training.

## CONCLUSION

As the world and the scientific community recover from the ongoing COVID-19 pandemic, there is ample opportunity to reflect on the importance of open science and open data. Open science and FAIR data depend on continuously evolving data resources and community-driven data standards and guidelines. The work carried out at EMBL-EBI during the past year underscores these principles, offering new data resources for emerging data types, new features and updates to the current portfolio of data resources, and work on establishing new data standards and guidelines to drive the quality of the data contained within databases.
